# Inhibited CSF1R Alleviates Ischemia Injury via Inhibition of Microglia M1 Polarization and NLRP3 Pathway

**DOI:** 10.1155/2020/8825954

**Published:** 2020-08-28

**Authors:** Xiaoxue Du, Yuzhen Xu, Shijia Chen, Marong Fang

**Affiliations:** ^1^Institute of Neuroscience, Zhejiang University School of Medicine, Hangzhou, 310006 Zhejiang, China; ^2^Translational Medicine Center, Affiliated Hangzhou First People's Hospital, Zhejiang University School of Medicine, Hangzhou, 310006 Zhejiang, China; ^3^Department of Neurology, Shanghai Tenth People's Hospital, Tongji University School of Medicine, Shanghai 200072, China

## Abstract

Ischemia cerebral stroke is one of the common neurological diseases with severe inflammatory response and neuron death. The inhibition of colony-stimulating factor 1 receptor (CSF1R) which especially expressed in microglia/macrophage exerted neuroprotection in stroke. However, the underlying neuroinflammatory regulation effects of CSF1R in ischemia stroke are not clear. In this study, cerebral ischemia stroke mice model was established. The C57/B6J mice were administered with Ki20227, a CSF1R inhibitor, by gavage for 7 consecutive days (0.002 mg/kg/day) before modeling. The Rota-Rod test and neurobehavioral score test were investigated to assess neurobehavioral functions. The area of infarction was assessed by 2, 3, 5-triphenyltetrazolium chloride (TTC) staining. The mRNA expressions of M1/M2 microglia markers were evaluated by real-time PCR. Immunofluorescence and Western blot were utilized to detect the changes of Iba1 and NLRP3 pathway proteins. Results showed that neurobehavioral function improvement was demonstrated by an increased stay time on the Rota-Rod test and a decreased neurobehavioral score in the Ki20227 treatment group. The area of infarction reduced in Ki20227 group when compared to the stroke group. Moreover, the mRNA expression of M1 microglia markers (TNF-*α* and iNOS) decreased while M2 microglia markers (IL-10 and Arg-1) increased. Meanwhile, compared to the stroke and stroke+PBS group, Ki20227 administration downregulated the expression of NLRP3, active caspase 1, and NF-*κ*B protein in the ischemia penumbra of Ki20227 treatment group mice. In short, the CSF1R inhibitor, Ki20227, played vital neuroprotective roles in ischemia cerebral stroke mice, and the mechanisms may be via inhibiting microglia M1 polarization and NLRP3 inflammasome pathway activation. Our study provides a potential new target for the treatment of ischemic stroke injury.

## 1. Introduction

Cerebral ischemic stroke remains the leading cause of the death and disability worldwide, despite progress in reperfusion therapies. Considerable empirical evidence has demonstrated that inflammatory response of microglia/macrophages to cerebral ischemia acts a vital part in varied phases of stroke pathobiology and outcome [1]. Excessive activation of proinflammatory cytokines, neural cell death, the blood-brain barrier, and neurogenesis disruption jointly lead to postischemic brain injury [2].

Colony-stimulating factor 1 receptor (CSF1R) is a class of tyrosine/serine kinases which especially expressed in microglia/macrophage and mainly responsible for regulating the proliferation and differentiation of microglia/macrophages [3]. Researches demonstrated the inhibition of CSF1R could regulate neurological function and exert neuroprotection in brain injury via inhibition of microglial activation [4]. Short-term elimination of microglia by CSF1R inhibitor, PLX5622, during the chronic phase of TBI disease led to long-term improvements in neurological function through decrease in NOX2 and NLRP3 inflammasome-associated neuroinflammation and improvement in the persistent neurodegenerative processes [5]. CSF1R inhibitor treatment significantly attenuates CSF1R activation and minimizes microglia cell proliferation with mRNA levels of proinflammatory factors and then enhances motor functions [6]. In global cerebral ischemia, Ki20227 as a specific inhibitor of CSF1R effectively protect the dendritic spine density and dendritic structure of neurons from brain injury [7]. And Ki20227 could inhibit the phosphorylation of CSF1R and played an important role in microglia activation [8]. However, the underlying neuroprotective mechanism of Ki20227 in brain ischemia injury is still unclear.

NOD-like receptor 3 (NLRP3) inflammasome plays an important role in neuroimmune responses including microglial-dependent activation. Regulating NLRP3 inflammatory response may be in favor of neurofunction improvement in ischemia stroke [9]. ATP reduction and rapid ROS increase and other diverse endogenous danger signals caused by ischemic injury of brain tissue could mediate the activation of the NLRP3 inflammasome in the pathophysiological process of ischemia stroke [10]. Immune cells including microglia could be recruited by sudden increase of inflammatory cytokines released after the NLRP3 inflammasome to clear the damage-associated molecular patterns (DAMPs) and affect microglia phenotypic M1/M2 transformation by initiating the inflammatory mechanisms after ischemia stroke. For example, high level of TNF-*α* released from the microglia M1 phenotype activation was disadvantaged in brain tissue and neuron recovery after stroke [11], while the microglia M2 factors exerted a neuroprotective role in various stages of acute ischemia stroke [12]. Microglial M1 inhibition and microglial M2 polarization activation could protect against ischemic stroke and have the potential to improve neurogenesis [13]. Meanwhile, several researches reported the NLRP3 inflammasome overactivation could give rise to secondary brain injury following reperfusion due to sustained inflammation release and brain damage aggravation which could be improved by the inhibition of NLRP3 inflammasome [14]. Studies demonstrated RNAi-mediated NLRP3 transcript knockdown decreased microglia-related inflammatory response and neuronal injury to alleviate brain injury and improve neurological outcomes in ischemia stroke [15]. However, the regulation mechanism of CSF1R inhibitor on microglia phenotype and NLRP3 pathway in ischemia stroke is still not clear. To address this question, we probed into the neuroinflammatory regulation role of Ki20227 in postischemic brain injury mice, and we elaborated the regulation of Ki20227 to NLRP3 pathway in mice subjected to cerebral ischemic injury.

## 2. Material and Methods

### 2.1. Animals

20–30 g weight C57BL/6 male mice were purchased from the Zhejiang Medical Academy. Before experiment, all animals were kept in pathogen-free separated clean cages with enough food and water under a constant temperature of 24°C and a 12 hours light/dark cycle. The Guide for Care and Use of Animal Center of Zhejiang University was followed, and the Ethics Committee for Use of Experimental Animals in Zhejiang University Animal treatment formally permitted animal treatment in this study.

### 2.2. Experimental Groups and Drug Administration

The fifth male C57BL/6 mice were stochastically split into 5 experimental groups: normal group (*n* = 10), sham group (*n* = 10), stroke group (*n* = 10), stroke+PBS group (*n* = 10), and stroke+Ki20227 group (*n* = 10). In stroke+Ki20227 group, Ki20227 (0.002 mg/kg/day dose, by gavage) was administered for 7 days [7]. After 7 days of Ki20227 administration, cerebral ischemia was established, and the Ki20227 administration was given once for the next 24 hours. Mice in stroke+PBS group underwent the same way of injection with equal volumes of PBS solution simultaneously.

### 2.3. Establishment of Cerebral Ischemic Mice Model

The cerebral ischemic model was established as previously described [16]. In short, Rose Bengal dye (Sigma-Aldrich, # 330000) was dissolved in 0.1 M PBS solution with a final concentration of 10 mg/mL, filtered through 0.45 m filters, and placed in the dark until use. At the beginning of the establishment of the model, Rose Bengal dye (100 mg/kg) was injected intraperitoneal. The mice were all anesthetized with inhaled isoflurane (RWD, China) when building the model. After 5 minutes, the target area (1.75 mm lateral to Bregma, +0.5 mm, left hemisphere) was exposed directly under the flexible cool light attached to a light-emitting diode cold light source (OPLENIC, M-IL-HAL 3001), and the continuous cool light irradiation was continued for 15 minutes to induce focal cerebral ischemia, and finally, the wound was sutured.

### 2.4. Behavioral Tests

#### 2.4.1. Rota-Rod Test

The Rota-Rod test was employed to measure the motor coordination and antifatigue ability of mice before and after cerebral ischemia treatment. The Rota-Rod test comprises a rotating rod with a diameter of 3 cm (this rod was divided into 5 tracks with a width of 6 cm), an infrared detector, and a computer. In the test, mice were positioned on the horizontally oriented, rotating rod. The infrared sensor sensed whether mice stayed on the stick, so as to obtain the time mice stayed on the rod and the rotation speed of the rod when they fell. The total test time was set to 300 seconds, and the rotation speed of rod was expedited from 8 rpm to 40 rpm. Each group mice were trained 3 times a day with a 10-minute rest each time. The mice staying on the rotating rod track for more than 200 seconds were used to establish the stroke model. The times of each group on the rotating rod instrument two days before the model establishment and one day after stroke were recorded.

#### 2.4.2. Neurobehavioral Score

The neurological scores were descripted previously [17]. There are 4 grades for neurobehavioral function evaluation. The higher score, the worse behavioral function. Number 4 represented unidirectional circling and consciousness reduction. Number 3 is unidirectional circling. Number 2 stands for forelimb flexion and resistance to lateral thrust reduction. Number 1 means forelimb flexion. Number 0 stands for no observable defects of mice. The above behavioral observation was performed in a blinded process. The score of each group was recorded at 24 h after stroke.

### 2.5. TTC Staining

Animals in different groups were sacrificed at 24 hours after building ischemia stroke model for brain infarct formation evaluation. TTC staining purchased from Sigma-Aldrich Company was used to measure the change of infract areas. Fresh brains were removed and kept at -20°C for 30 min and then were sliced into 2 mm coronal sections rapidly. 5% TTC in physiological saline solution was used to incubate every slice at 37°C for 20 min in dark. And then 4% buffered formalin fixed the slice for 1 hour at room temperature. The infract areas were showed as the complexionless areas and presented in the figures. Infarct areas were measured using ImageJ software (NIH, Bethesda, MD, USA). The total infarction volume for each slice was calculated. The infarction volume of every mouse was calculated from the infarcted area of the ipsilateral hemisphere/total area (from both the ipsilateral and contralateral hemispheres) [17].

### 2.6. Immunofluorescence Staining

Brains of every group mouse were perfusion by 0.9% saline solution and fixed in 4% paraformaldehyde PBS solution at 4°C overnight. After dehydrated in 30% sucrose solution for one week, brains were sectioned coronally with embedding them with OCT. Rabbit anti-Iba1 (1 : 500; Abcam, #ab178847) and rabbit polyclonal to anti-NLRP3 (1 : 100; BOSTER, BM4490) as primary antibodies were applied. Secondary antibody including goat anti-rabbit Alexa Fluor 488 (1 : 500, EARTHOX, San Francisco, # E031220-01) was employed after primary antibodies. Before being covered with coverslips, the slides were added the antiquenching solution with DAPI dye (VECTASHIELD, USA) for observation. Images were taken in 5-vision/section stochastically to count the immunoreactive cells under Olympus BX51 fluorescence microscope [15].

### 2.7. Western Blot

Total proteins of ischemic brain tissue in each group were extracted by RIPA buffer containing EDTA-free protease inhibitor and phosphate inhibitor (Rocher, Switzerland). After measuring the protein concentration, 30 *μ*g of each sample was subjected to electrophoresis on 15% SDS-PAGE gel at 200 V. The protein was transferred into the PVDF membrane at 100 V in Bio-Rad TransBlot apparatus. 5% skimmed milk diluted by TBST solution was used to block PVDF membranes containing proteins for 2.5 hours at room temperature, and then, the primary antibody, rabbit anti-GADPH (1 : 1000, Abcam, ab181602), NLRP3 (1 : 1000, Abcam, ab181602), caspase 1 (1 : 1000, Abcam, ab181602), and NF-*κ*B (1 : 1000, Abcam, ab181602), were used to incubate PVDF membranes at 4°C overnight. And then, the second antibody HRP-conjugated goat anti-rabbit (1 : 5000; EarthOx, USA) was used to incubate membranes for 2.5 hours after washing three times per 5 mins with TBST solution. Finally, after incubating with enhanced chemiluminescence, the membranes were exposed in ChemiDoc Touch Imaging System. ImageJ software was applied to the quantification analysis of protein bands. Each experiment was performed three times [16].

### 2.8. Real-Time PCR

After extracting total RNA with Trizol (Invitrogen) reagent, it was reverse transcribed into complementary deoxyribonucleic acid (cDNA) according to the instructions of BestarTM qPCR RT kit (DBI-2220, Germany). Real-time PCR protocol was carried out under the guidance of BestarR SybrGreen qPCR master mix (DBI-2044, Germany). The results were interpreted in Bio-Rad CFX manager program 3.0 software. The microglia M1/M2 type factor primers are shown in Table 1.

### 2.9. Statistical Analysis

SPSS 22.0 software was used to perform statistical analysis. The one-way ANOVA with Tukey test evaluated the difference between the drug treatment and nondrug treatment group in stroke mouse models. For the data of multiple groups in the Rota-Rod test, the difference was analyzed by the two-way ANOVA with Bonferroni posttest. Values are presented as means ± SEM. For all of the tests, three levels of significance were determined: ^∗^*P* < 0.05, ^∗∗^*P* < 0.01, ^∗∗∗^*P* < 0.001.

## 3. Results

### 3.1. Ki20227 Administration Reduced Neurological Deficits

**Figure 1:**
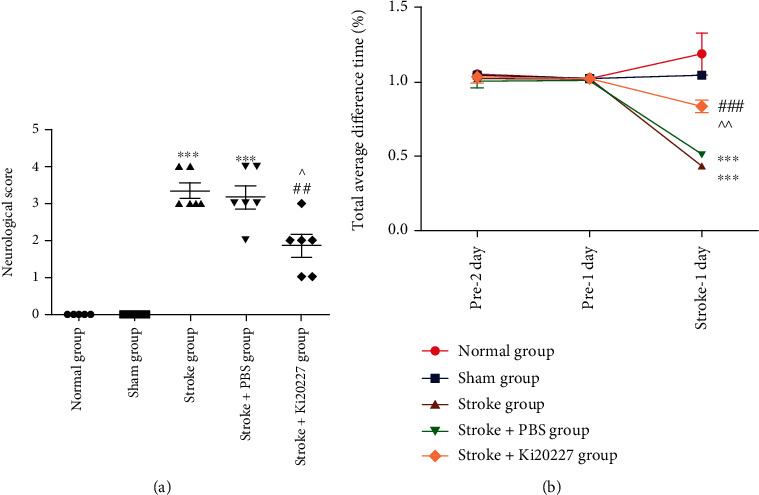
Ki20227 administration reduced neurological deficits. Statistical analysis of behavior tests. Statistical analysis of (a) neurobehavioral score and (b) the total average difference time in Rota-Rod test for normal, sham, stroke, stroke+PBS, and stroke+Ki20227 groups. Normal group vs. stroke group, ^∗∗∗^*P* < 0.001; normal group vs. stroke+PBS group, ^∗∗∗^*P* < 0.001; stroke+PBS vs. stroke+Ki20227 groups, ^*P* < 0.05, ^^*P* < 0.01; stroke+Ki20227 group vs. stroke group, ^##^*P* < 0.01, ^###^*P* < 0.001.

Neurological scores and Rota-Rod test were classical methods and used to evaluate the neurobehavioral damage in each group. The increase neurological deficit score or the decrease time in Rota-Rod indicated the severe impairment of motor function of mice. Results showed animals in the stroke group and stroke+PBS group had the increased neurological score indicating more neurological deficits when compared to the sham group (^∗∗∗^*P* < 0.001) in Figure 1(a). However, Ki20227 pretreated could significantly decrease neurological score to improve neurological impairment in the stroke+Ki20227 group when compared to the stroke and stroke+PBS groups (^##^*P* < 0.01, ^*P* < 0.05). In the Rota-Rod test of Figure 1(b), animals in every group showed the average different time was close to 1 in pre-1 day and 2 days. After building stroke model, the average different time of animals in the stroke group and stroke+PBS group on the Rota-Rod significantly less than that of the normal and sham groups (^∗∗∗^*P* < 0.001). Meanwhile, mice in stroke+Ki20227 group had significantly more time on the Rota-Rod than that of the stroke and stroke+PBS groups (^###^*P* < 0.001, ^^*P* < 0.01). And there is no significant difference between stroke group and stroke+PBS group considering the results in the behavioral tests.

### 3.2. Ki20227 Administration Reduced the Brain Infarction Volume

**Figure 2:**
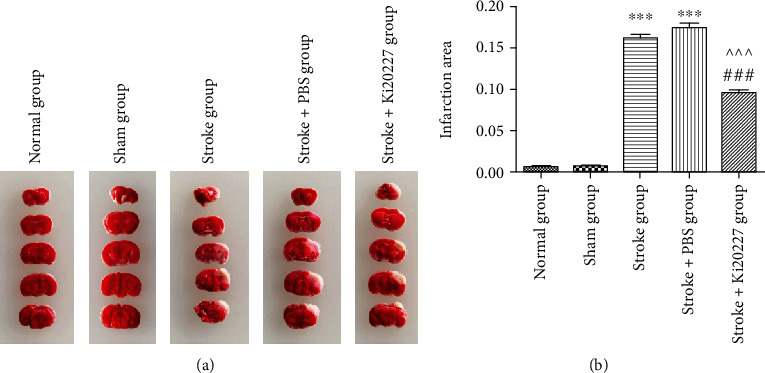
Ki20227 administration reduced the brain infarction volume. (a) The representative images of TTC staining in each group. (b) Statistic analysis of infarction volume in normal, sham, stroke, stroke+PBS, and stroke+Ki20227 groups. Normal group vs. stroke group, ^∗∗∗^*P* < 0.001; normal group vs. stroke+PBS group, ^∗∗∗^*P* < 0.001; stroke+Ki20227 group vs. stroke group, ^###^*P* < 0.001; stroke+PBS vs. stroke+Ki20227 groups, ^^^*P* < 0.001.

The infarct volume of brain sections of every group is analyzed by TTC staining after 24 hours of the ischemic insult. In stroke and stroke+PBS groups, the infarct volume was significantly increased compared to that of normal and sham groups (^∗∗∗^*P* < 0.001). There was no significant difference in the infarction volume between the stroke and stroke+PBS groups (Figures 2(a) and 2(b)). However, compared to the stroke and stroke+PBS groups, Ki20227 treatment reduced the infarct volume of stroke+Ki20227 group mice (^###^*P* < 0.001, ^^^*P* < 0.001).

### 3.3. Ki20227 Administration Reduced Iba1 Expression after Stroke

**Figure 3:**
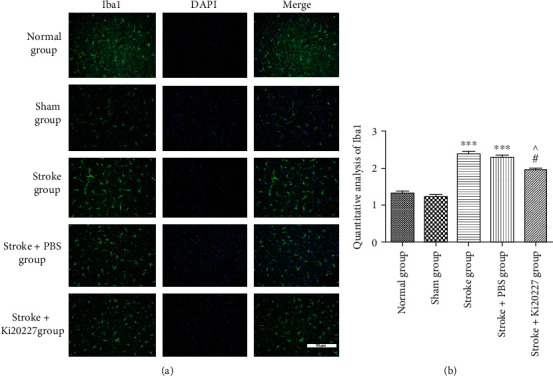
Ki20227 administration reduced Iba1 expression after stroke. (a) In normal, sham, stroke, stroke+PBS, and stroke+Ki20227 groups, Iba1 protein immunofluorescence staining was showed into green fluorescence. Blue fluorescence represented and was labeled as the cell nucleus. Merged pictures are showing positive cells. Bar = 50 *μ*m. (b) Statistical analysis results of Iba1 expression in each group. The IOD/area of proteins expression assumed the comparative expression. Normal group vs. stroke group, ^∗∗∗^*P* < 0.001; normal group vs. stroke+PBS group, ^∗∗∗^*P* < 0.001; stroke+Ki20227 group vs. stroke group, ^#^*P* < 0.05; stroke+PBS vs. stroke+Ki20227 groups, ^*P* < 0.05.

Iba1 immunofluorescent analysis was used to investigate the rest and activation of microglia in the penumbral area of stroke mice. As shown in Figure 3, in the stroke group and stroke+PBS group, Iba1 expression is significantly more than that of the sham group (^∗∗∗^*P* < 0.05). However, Iba1 expression significantly decreased in stroke+Ki20227 group, when compared with stroke and stroke+PBS group (^#^*P* < 0.05, ^*P* < 0.05). Results demonstrated Ki20227 could inhibit the Iba1-positive microglia in ischemia stroke model mice.

### 3.4. Ki20227 Administration Inhibited Microglia M1 Phenotype and Activated M2 Phenotype

**Figure 4:**
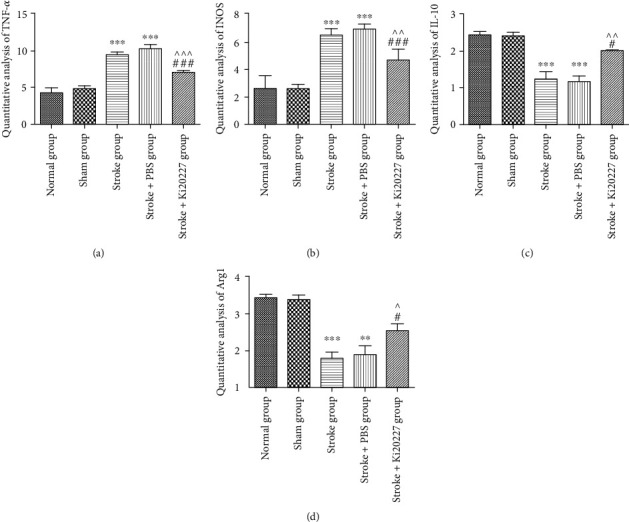
Ki20227 administration inhibited microglia M1 phenotype and activated M2 phenotype. Real-time PCR statistical analysis of (a) TNF-*α*, (b) iNOS, (c) IL-10, and (d) Arg-1 for normal, sham, stroke, stroke+PBS, and stroke+Ki20227 groups. Normal group vs. stroke group, ^∗∗∗^*P* < 0.001; normal group vs. stroke+PBS group, ^∗∗∗^*P* < 0.001; stroke+Ki20227 group vs. stroke group, ^#^*P* < 0.05, ^##^*P* < 0.01; stroke+PBS vs. stroke+Ki20227 groups, ^*P* < 0.05, ^^*P* < 0.01, ^^^*P* < 0.001.

To further explore the anti-inflammation effects of Ki20227, microglia M1 and M2 factors expression in the penumbra of stroke mice were illuminated by real-time PCR as shown in Figure 4. The low levels of microglia M1 phenotype (TNF-*α* and iNOS) were revealed in the brain of sham group mice (Figures 4(a) and 4(b)). After building stroke model, the mRNA levels of TNF-*α* and iNOS of penumbra of stroke and stroke+PBS groups were markedly upregulated (^∗∗∗^*P* < 0.001). In contrast, Ki20227 treatment could significantly decrease the mRNA expression of TNF-*α* and iNOS based on building stroke model with PBS treatment (*^##^P* < 0.01, *^###^P* < 0.001, *^^P* < 0.01, *^^^P* < 0.001). Meanwhile, the mRNA expression of microglia M2 phenotype factors with anti-inflammatory effects was clarified to further explore the neuroprotection role of Ki20227. In Figures 4(c) and 4(d), the low mRNA expression of Arg-1 and IL-10 as microglia M2 phenotype was observed in the penumbra of sham group mice. The decrease mRNA levels of Arg-1 and IL-10 were markedly showed in stroke and stroke+PBS groups and less than that of the sham group (^∗∗^*P* < 0.01, ^∗∗∗^*P* < 0.001). However, Ki20227 administration significantly increased Arg-1 and IL-10 mRNA expression in Ki20227+stroke group when compared to stroke and stroke+PBS groups (^#^*P* < 0.05, ^###^*P* < 0.001, ^*P* < 0.05, ^^*P* < 0.01).

### 3.5. Ki20227 Administration Inhibited NLRP3 Pathway Activation

**Figure 5:**
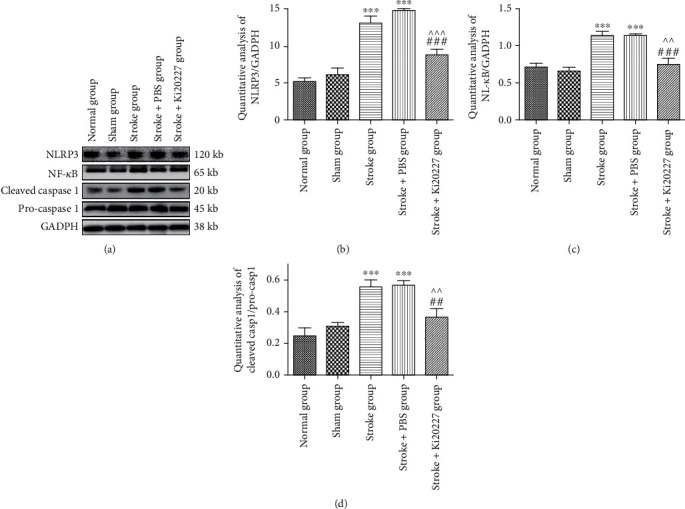
Ki20227 administration inhibited NLRP3 pathway activation. Western blot of (a) NLRP3, NF-*κ*B, cleaved caspase 1, pro-caspase 1, and GADPH for normal, sham, stroke, stroke+PBS, and stroke+Ki20227 groups. Statistical analyses of (b) NLRP3, (c) NF-*κ*B, and (d) cleaved caspase 1/pro-caspase 1. Normal group vs. stroke group, ^∗∗∗^*P* < 0.001; normal group vs. stroke+PBS group, ^∗∗∗^*P* < 0.001; stroke+Ki20227 group vs. stroke group, ^##^*P* < 0.01, ^###^*P* < 0.001; stroke+PBS vs. stroke+Ki20227 groups, ^^*P* < 0.01, ^^^*P* < 0.001.

We further investigated the expression changes of NLRP3 signaling pathway proteins caused by ischemic injury. NLRP3 inflammatory response is associated with microglia activation which maybe contributed to the neuron recovery after stroke though NF-*κ*B and caspase 1 activation. Western blot results showed the low protein expression of NLRP3 in sham and normal groups in Figure 4. Compared with sham and normal groups, the NLRP3, NF-*κ*B, and cleaved caspase 1 protein expression increased significantly in both stroke group and stroke+PBS group (^∗∗∗^*P* < 0.001). In contrast, Ki20227 treatment could decrease the NLRP3, NF-*κ*B, and cleaved caspase 1 protein expression in stroke+Ki20227 group after building stroke model with PBS treatment (^##^*P* < 0.01, ^###^*P* < 0.001, ^^*P* < 0.01, ^^^*P* < 0.001) in Figures 5(b)–5(d).

### 3.6. Ki20227 Administration Inhibited NLRP3 Inflammasome Activation

**Figure 6:**
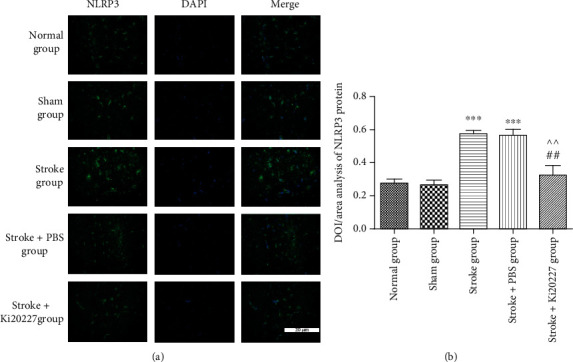
Ki20227 administration inhibited NLRP3 inflammasome activation. (a) NLRP3 protein in immunofluorescence staining is labeled with green fluorescence in normal, sham, stroke, stroke+PBS, and stroke+Ki20227 groups. Blue fluorescence represented and was labeled as cell nucleus. Positive cells are showed in merged pictures. Bar = 20 *μ*m. (b) Results of Iba1 protein expression statistical analysis. Normal group vs. stroke group, ^∗∗∗^*P* < 0.001; normal group vs. stroke+PBS group, ^∗∗∗^*P* < 0.001; stroke+Ki20227 group vs. stroke group, ^##^*P* < 0.01; stroke+PBS vs. stroke+Ki20227 groups, ^^*P* < 0.01.

Meanwhile, the NLRP3 immunofluorescent analysis was implemented to examine NLRP3 inflammasome in the penumbral area of stroke mice as displayed in Figure 6. Results revealed NLRP3 inflammasome was activated in the ischemic brain of stroke and stroke+PBS groups with high NLRP3 protein expression (^∗∗∗^*P* < 0.001). Ki20227 administration significantly prevented ischemia-induced NLRP3 expression when compared to stroke and stroke+PBS groups (^##^*P* < 0.01, ^^*P* < 0.01).

## 4. Discussion

**Figure 7:**
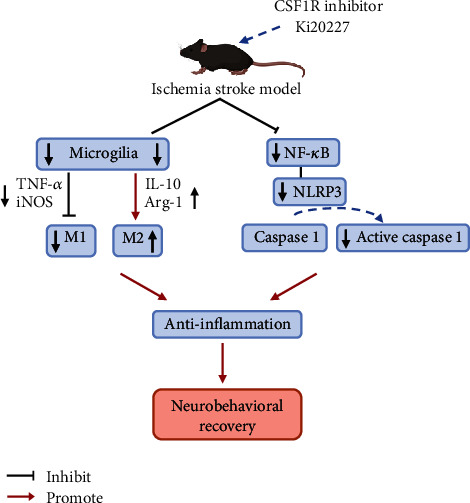
Illustrative mechanism for Ki20227 administration to alleviate the ischemic injury. Ki20227 treatment as CSF1R inhibitor could inhibit microglia number and improve behavioral deficits and attenuate microglia-related inflammation, which is verified by a reduction in both microglia M1 phenotype activation and Iba1 expression, meanwhile, increase of microglia M2 phenotype expression. The downregulation of the NLRP3 pathways and inflammasome activation involved the neuroprotective mechanism of CSF1R inhibitor in ischemia stroke.

In the present study, our results indicated that the Ki20227, as CSF1R inhibitor administration, could improve ischemia-induced behavioral deficits of mice and attenuate microglia-related inflammation, which is validated by the downregulation of mRNA expression in both microglia M1 phenotype factors and Iba1 protein expression, meanwhile, increase of microglia M2 phenotype expression shown as in Figure 7. The inhibition of NLRP3 pathway involved the neuroprotective mechanism of Ki20227 in ischemia stroke mice.

Once stroke occurs, the disruption of neural circuits including dysfunction between the different cell types could destructively affect the learning, memory, sensory, and motor abilities of mice [18]. Evaluating the behavioral performance of mice after stroke could reflect neural function injury and recovery. Meanwhile, reported data showed the upregulation of microglial-related protein is closely associated with repetitive behavior, social deficits, and synaptic dysfunction of mice. Treatment with colony-stimulating factor 1 receptor inhibitors that induced microglial depletion and repopulation could correct behavioral abnormalities by modulating neurogenic molecules in microglia [19]. Our results showed Ki20227 has no influence on the behavioral function when compared to normal group before building stroke model in Rat-Rot test. Meanwhile, animals showed significantly less time on the Rota-Rod test and higher neurological scores followed stroke. Using Ki20227 inhibitor in mice has an efficient effect to improve the behavioral function which is obviously showed through increasing the time on Rota-Rod and reducing behavioral defect score as well as decreasing Iba1 expression.

Microglial activation was initiated from hours after stroke and could develop in the next couple of days. The therapeutic intervention is presented with a much longer window for neuroprotection [20]. Some evidence demonstrated that the inflammation including microglia activation and the release of cytokines and trophic factors could reach the peak and exert significant role in the first few days after stroke [21]. In our previous studies, intracellular signal pathway including autophagy and inflammation significantly dysregulated at 24 hours after building middle cerebral artery occlusion (MCAO) model and could be revised by the drug administration. For example, triptolide exerted neuroprotection in cerebral ischemia stroke through downregulating NF-*κ*B- and iNOS-induced inflammation and upregulating autophagy at the 24-hour timepoint after building MCAO model [17, 22]. In this study, we also did the detail experiment at the 24-hour timepoint after building stroke model. Meanwhile, microglia-related inflammatory responses take a critical part in the pathological processes of ischemia injury including microglia phenotype transformation and inflammatory factors release [1]. Numerous works indicated that activating microglia M1 phenotype response has a negative effect on the process of cerebral ischemia [23]. Inhibiting microglia M1 phenotype and activating M2 phenotype have a broad prospect in treating stroke [24]. In ischemic brain injury, the inflammatory response can prompt the massive release of microglia M1 factors such as TNF-*α* and iNOS [25]. Overexpression of TNF-*α* can aggravate brain injury, while the upregulation of iNOS expression will increase the nitric oxide (NO) synthesis and secretion, which in turn promotes the aggregation of microglia to the injury site. As a result, a toxic effect on the nervous system and an exacerbated brain injury might be occurred. Overexpression of S100B which is expressed in microglia could aggravate cerebral ischemia through activating NF-*κ*B expression and inhibiting M2 stimuli expression to promote microglia M1 polarization [26]. Inhibited microglia M1 and activated M2 polarization by treating with curcumin and dihydrolipoic acid-gold nanoclusters prevents ischemic stroke and has the potency to ameliorate neurogenesis [27]. In this study, the expression of TNF-*α* and iNOS mRNA induced by focal cerebral ischemic injury was increased significantly, and the number of microglial cells at the injured site increased as well. The Ki20227 drug could effectively reduce Iba1 expression and inhibit microglia M1 factor expression (i.e., TNF-*α* and iNOS) expression. In the same manner, activating microglia M2 factor expression could improve the neuron connection of stroke mice along with reductions in elevated proinflammatory molecules and standardization of synaptophysin and PSD-95 expression [28, 29]. Moreover, IL-4 as cytokine anti-inflammatory factor could induce microglia M2 polarization including increasing IL-10, TGF-*β*, YM1, Arg-1, and IGF-1 secretion. This role might facilitate the reforming and regeneration of neurons through GSH and NGF upregulation and apoptosis inhibition [30]. Results showed a significant decrease of IL-10 and Arg-1 expression after ischemic injury of mice; meanwhile, the number of microglial cells at the injury site was increased. This implies that the Ki20227 drug can effectively increase microglia M2 factor IL-10 and Arg-1 expression.

NLRP3 inflammasome participated critical part in neuroimmune response including microglial-dependent activation [31]. This protein can be induced by various endogenous and exogenous danger signals and subsequently activate caspase 1. This action may promote the maturation and release of IL-1*β* and IL-18 [32]. In this regard, several previous studies have found that the activation of NLRP3 could increase the expression of inflammatory genes including NF-*κ*B and TNF-*α*, which consisted with our results [33]. Recent findings demonstrated that NLRP3 inflammasome play a crucial role in regulating inflammatory responses in the pathological process of ischemic stroke. Not only that a growing number of studies have shown that the NLRP3 suppression serves as neuroprotective role in ischemia stroke through alleviating infarction volumes and neurovascular damages to improve ischemia outcomes [34]. Inhibited NLRP3 proteins using special antagonist attenuate brain injury and inflammation after hemorrhagic stroke [35]. Liu and his research team found that the NLRP3 inflammasome inhibitor provides a neuroprotection effect in stroke through TLR4/NF-*κ*B/NLRP3 signaling pathway [36]. Genetic modulations of NLRP3 impression in the stroke animal model bring out significant protection against microglia-related inflammatory responses [37]. Moreover, gene silencing of microglia P2X7R or TMEM proteins reduced NLRP3 activation to attenuate brain edema and neurological deficits [38]. The link between microglia inflammatory signaling and NLRP3 activation was indicated by our results. CSF1R inhibitor could reduce the mRNA expression of TNF-*α* and protein expression of NF-*κ*B and activate caspase 1 to inhibit the NLRP3 inflammasome activation.

The more widely held view is that the CSF1R signaling or CSF1R-dependent microglial signaling suppression would perform protective function for several diseases including cancer therapy, neurodegenerative disease, and ischemia stroke [39, 40]. A randomised phase 3 trial for tenosynovial giant cell tumour with aberrantly expressing colony-stimulating factor 1 (CSF1) showed pexidartinib as CSF1R inhibitor could improve patient symptoms and functional outcomes [41]. The underlying mechanism was indicated as the proinflammatory function activation of CSF1R signaling; however, CSF1R blockade antibody significantly minificated the inflammatory reaction and alleviated disease related symptoms [42]. In neurodegenerative disease, CSF1R signaling might activate proinflammatory processes through regulating CSF1 and IL34 signaling [43]. Inhibition of CSF1R signaling may provide an intervention approach or therapeutic due to the inhibition of the process of amyloid deposition formation at the very earliest stages of Alzheimer's disease [44]. It may be related with CSF1 participated in ameliorated memory deficits and regulated IL34 release to significantly reduce excitotoxic-induced neuronal loss [45]. In ischemia stroke, CSF1R expression upregulated with neuron death and behavioral deficit. Our results indicated Ki20227 exerted neuroprotective via improving ischemia-induced behavioral deficits and reducing the infarction volume. The inhibition of NLRP3 pathway and microglia-related factor release reduction could prove the neuroprotective role of CSF1R. The precise mechanism was also revealed that microglial density and survival were dependent on CSF1R signaling and inhibition of CSF1R in microglia contributed to the dendritic spines and neurons recovery in ischemia stroke [29].

Taken together our findings, Ki20227 takes the neuroprotective and anti-inflammatory roles via inhibiting microglia M1 phenotype and activating M2 polarization under NLRP3 inflammasome and NLRP3 signaling pathway inhibition in focal cerebral ischemia mice model. Hence, Ki20227 holds a promise as a promising drug target for clinical acute ischemia stroke patient therapy in the future.

## 5. Conclusion

The acute inflammatory reaction especially in microglia-mediated pathways displays an important role in regulating pathology processing after stroke. No theory has suggested a single pathway underlying stroke neuropathology to account for CSF1R inhibitor regulation on microglia. Besides, the effect of inhibited microglia in stroke is undefined. Our findings support that Ki20227 made neuroprotection through downregulating microglia M1 phenotype and NLRP3 pathways with microglia M2 phenotype activation to improving neuron behavioral recovery in stroke. Inhibited microglia CSF1R factor is indispensable in ischemic stroke to validate the evaluation of CSF1R inhibitors in clinical trials for ischemic brain diseases.

## Figures and Tables

**Table 1:** Sequence of primer of microglia M1 and M2 type factors.

Gene name	Forward primer (5′-3′)	Reverse primer (5′-3′)
TNF-*α*	GATCGGTCCCCAAAGGGATG	CCACTTGGTGGTTTGTGAGTG
Arg-1	CATGGGCAACCTGTGTCCTT	TCCTGGTACATCTGGGAACTTTC
IL-10	GTCATCGATTTCTCCCCTGTG	CCTTGTAGACACCTTGGTCTTGG
iNOS	TGGTGAGGGGACTGGACTTT	CCAACTCTGCTGTTCTCCGT
*β*-Action	CGTGCGTGACATCAAAGAGAAG	CAAGAAGGAAGGCTGGAAAAGA

Arg: arginase-1; TNF-*α*: tumor necrosis factor-*α*; IL-10: interleukin 10.

## Data Availability

The data used to support the findings of this study are available from the corresponding author upon reasonable request.
